# Disruption of a Rice Chloroplast-Targeted Gene *OsHMBPP* Causes a Seedling-Lethal Albino Phenotype

**DOI:** 10.1186/s12284-020-00408-1

**Published:** 2020-07-25

**Authors:** X. Liu, P. H. Cao, Q. Q. Huang, Y. R. Yang, D. D. Tao

**Affiliations:** 1 Key Laboratory of Eco-Agricultural Biotechnology around Hongze Lake, Regional Cooperative Innovation Center for Modern Agriculture and Environmental Protection, Huaiyin Normal University, Huaian, 223300 China; 2 Suzhou Academy of Agricultural Sciences, Suzhou, 215155 China

**Keywords:** Chloroplast development, CRISPR/Cas9, Rice (*Oryza sativa* L.), Albino lethal, RNA editing

## Abstract

**Background:**

Chloroplast development is coordinately regulated by plastid- and nuclear-encoding genes. Although many regulators have been reported to be involved in chloroplast development, new factors remain to be identified, given the complexity of this process.

**Results:**

In this study, we characterized a rice mutant *lethal albinic seedling 1*(*las1*)form of a 4-hydroxy-3-methylbut-2-enyl diphosphate reductase (OsHMBPP) that was targeted to the chloroplasts. The *LAS1* mutation caused the albino lethal phenotype in seedlings. Transmission electron microscopy indicated that *las1* were defective in early chloroplast development. *LAS1* is preferentially expressed in leaves, implying its role in controlling chloroplast development. The expression levels of many chloroplast-encoded genes were altered significantly in *las1*. The expression levels of nuclear-encoded gene involved in Chl biosynthesis were also decreased in *las1*. We further investigated plastidic RNA editing in *las1* and found that the edit efficiency of four chloroplast genes were markly altered. Compared with WT, *las1* exhibited defective in biogenesis of chloroplast ribosomes.

**Conclusions:**

Our results show that *LAS1/OsHMBPP* plays an essential role in the early chloroplast development in rice.

## Background

Chloroplasts are essential for plant development and growth, through manipulating the fixation of CO_2_ and biosynthesis of carbon skeletons and other physiological processes (Jarvis and López-Juez 2013). Accumulating evidence has shown the importance of chloroplast biogenesis and development during germination for plant vitality, seed set and growth (Lopez-Juez and Pyke 2005; Pogson and Albrecht 2011). Chloroplast biogenesis is initiated from proplastids through endosymbiosis from an ancestor of extant cyanobacteria and is dependent on the coordinated expression of genes encoded in both nuclear and plastid genomes (Lopez-Juez and Pyke 2005; Kessler and Schnell 2009). The development of chloroplasts differs between organs and species depending on the specialization of tissues and stage of development. For example, distinct phenotypes between cotyledons and true leaves were observed in *variegated* (*var*) and *snowy cotyledon* (*sco*) mutants in *Arabidopsis*, respectively (Albrecht et al. 2008; Liu et al. 2010; Zagari et al. 2017), i.e. chlorotic true leaves but green cotyledons in the var. and chlorotic or bleached cotyledons but green true leaves in the *sco* mutants. It is evident that nucleus-encoded polymerases (NEPs) and plastid-encoded polymerases (PEPs) involved in gene transcription, RNA maturation, protein translation and modification have great effect on the biogenesis of chloroplasts (Pogson and Albrecht, 2011; Yu et al. 2014).

Isoprenoids play essential roles in plant growth and development. In higher plants, biosynthesis of the basic isoprenoid units occurs by two different pathways: the mevalonate (MVA) pathway and the methyl-D-erythritol-4-phosphate (MEP) pathway. For decades, it was thought that the MVA pathway was solely responsible for isoprenoid biosynthesis. However, the alternative MEP pathway was discovered recently in bacteria, green algae, and higher plants (Rohmer et al. 1993; Cunningham Jr. et al. 2000; Rodriguez-Concepcion and Boronat, 2002; Rohmer, 2003). The MVA pathway occurs in the cytosol and is responsible for the synthesis of sterols, certain sesquiterpenes, and the side chain of ubiquinone (Disch et al. 1998). By contrast, the MEP pathway operates in the plastids and is involved in providing the precursors for monoterpenes, isoprene, chlorophylls, carotenoids, tocopherols, taxadiene, gibberellins, and abscisic acid (Zeidler et al. 1997; Eisenreich et al. 1998; Lichtenthaler, 1999). In recent years, the entire MEP pathway and almost all the enzymes involved have been identified in *Escherichia coli*. The activity of the corresponding enzyme and the roles they played in the pathway have been widely demonstrated (Lois et al. 1998; Herz et al. 2000; Hecht et al. 2001; Adam et al. 2002). The genes encoding each enzyme in the MEP pathway are highly conserved. Genes encoding enzymes involved in the MEP pathway are important for chlorophyll and carotenoid biosynthesis in plants. Knockout mutations of several genes in this pathway in *Arabidopsis, Zea may* and *Nicotiana benthamiana* display the albino phenotype (Budziszewski et al. 2001; Gutierrez-Nava et al. 2004; Page et al. 2004; Hsieh and Goodman, 2005; Lu et al. 2012). However, the genes involved in the MEP pathway in rice has not been reported yet and needs further study.

In this study, we obtained a rice mutant *lethal albinic seedling 1* (*las1*) by the CRISPR/Cas9 system. Identification of CRISPR/Cas9 sites revealed sequence differences in the gene encoding a 4-hydroxy-3-methylbut-2-enyl diphosphate reductase, which is involved in the isoprenoids biosynthesis. The mutant seedlings were albinic and eventually died from lacking of chlorophyll. Additionally, the transcript levels of genes associated with chlorophyll biosynthesis, and those associated with chloroplast biogenesis were severely affected in the *las1* mutant. These findings implicate that rice *LAS1* plays an important role in chloroplast biogenesis during early leaf development.

## Results

### Characterization of the *las1* Mutant in Rice

**Fig. 1:**
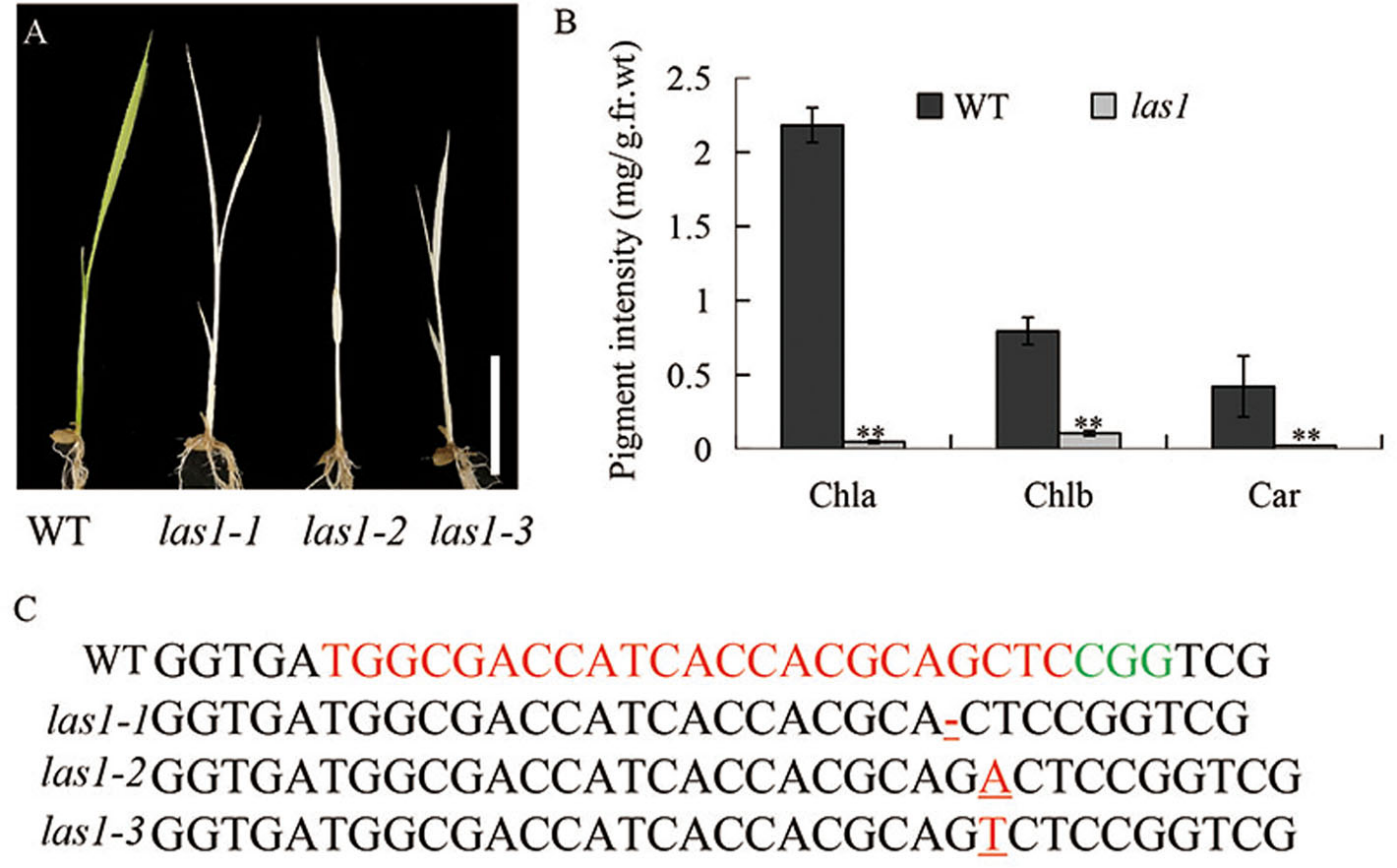
The phenotype and pigment contents of wild type and *las1*. **a** Morphology of wild type (WT) and *las1* seedling (Bar = 5 cm). **b** Pigment contents of WT and *las1* seedlings. Values represent the mean ± SD of three biological replicates. **p < 0.01. **c** Sequence confirmation of the *LAS1* mutation. The 23-nt target sequence of the Cas9/sgRNA complex is underlined in red

We isolated a rice mutant by the CRISPR/Cas9 system, which exhibited defects in leaf color, termed *lethal albinic seedling 1*(*las1*). The *las1* mutant exhibited the albino phenotype and eventually died (Fig. 1a; Additional file 1: Figure S1). Consistent with their phenotype, the chlorophyll contents were significantly reduced in *las1* compared with that of the wild type (Fig. 1b). Sequence confirmation indicated that the phenotype of *las1* resulted from the *LOC_Os03g52170* mutation encoding a 4-hydroxy-3-methylbut-2-enyl diphosphate reductase, OsHMBPP (Fig. 1c). We identified five homozygous mutants and the phenotype of the heterozygous mutants were similar to that of WT (Additional file 1: Figure S1). Protein sequence alignments of the homozygous mutants and the wild type protein revealed that three mutants showed premature translational stops, and the line *las1–1* encodes the least amino acids. So we chose the line *las1–1* to represent the *LAS1* mutant for further study. These data suggested that *LAS1/OsHMBPP* is essential for early chloroplast development in rice.

### Chloroplast Ultrastructure of the *las1* Mutant

**Fig. 2:**
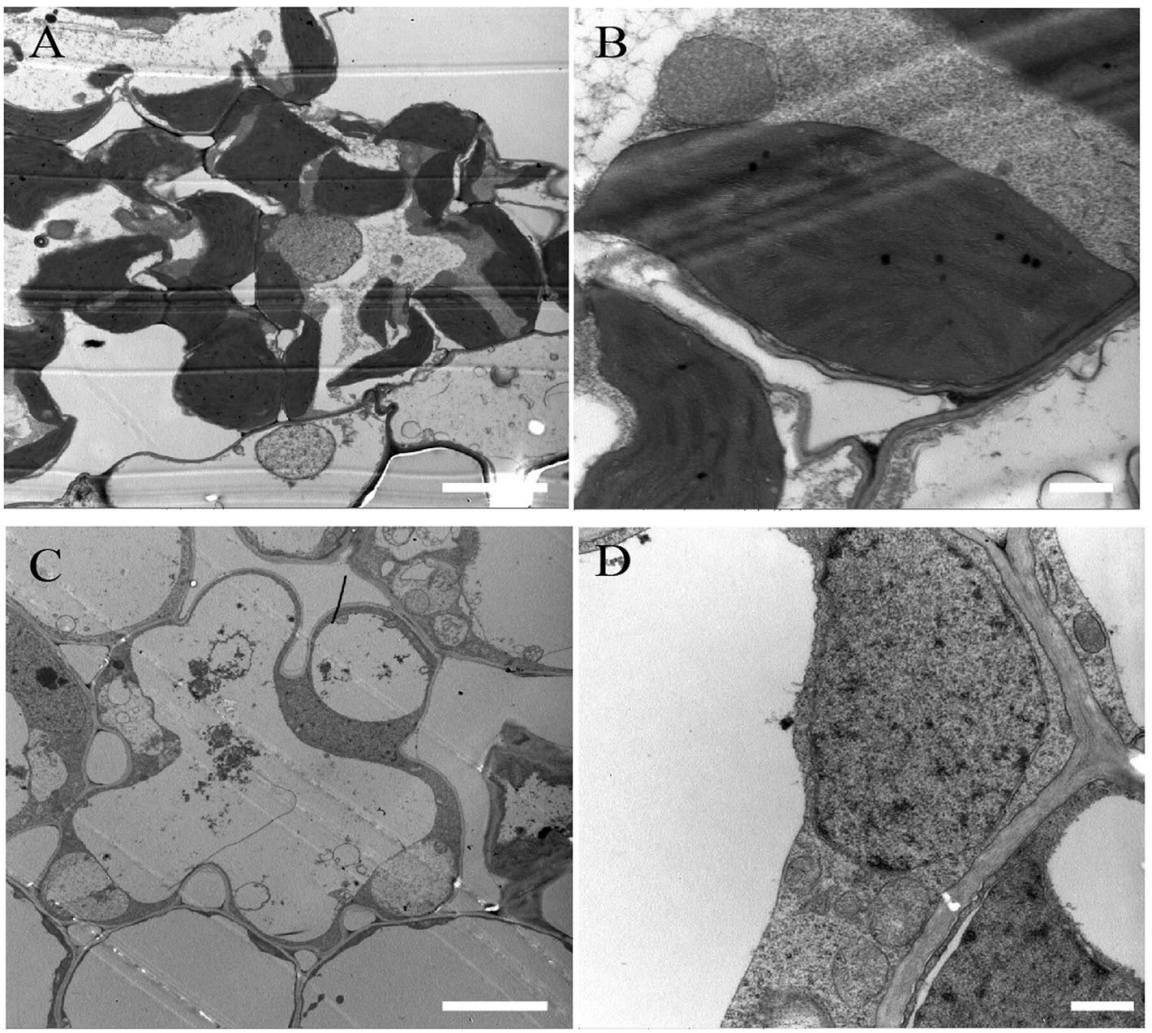
TEM analysis of chloroplast ultrastructure in wild type and the *las1* mutant. **a**-**b** Chloroplast structure in WT cell. **c**-**d** Chloroplast structure in *las1* cell. Scale bars 1 μm for (**b**) and (**d**); 5 μm for (**a**) and (**c**)

Chloroplast development depends on the successive synthesis and integration of Chl into photosynthetic complexes (Wang & Grimm, 2015). To further investigate the albino leaf phenotype of *las1*, we observed the chloroplast structure of *las1* and wild type in leaves by TEM. The chloroplasts of the WT leaves were normal in shape, containing large starch granules and normal thylakoid membranes with stacks of grana (Fig. 2a-b). In contrast, chloroplasts were severely disrupted in the *las1* leaves (Fig. 2c-d). Our results indicated that the albino leaf phenotype of *las1* resulted from the abnormal development of chloroplasts.

### Expression Pattern of *OsHMBPP*

**Fig. 3:**
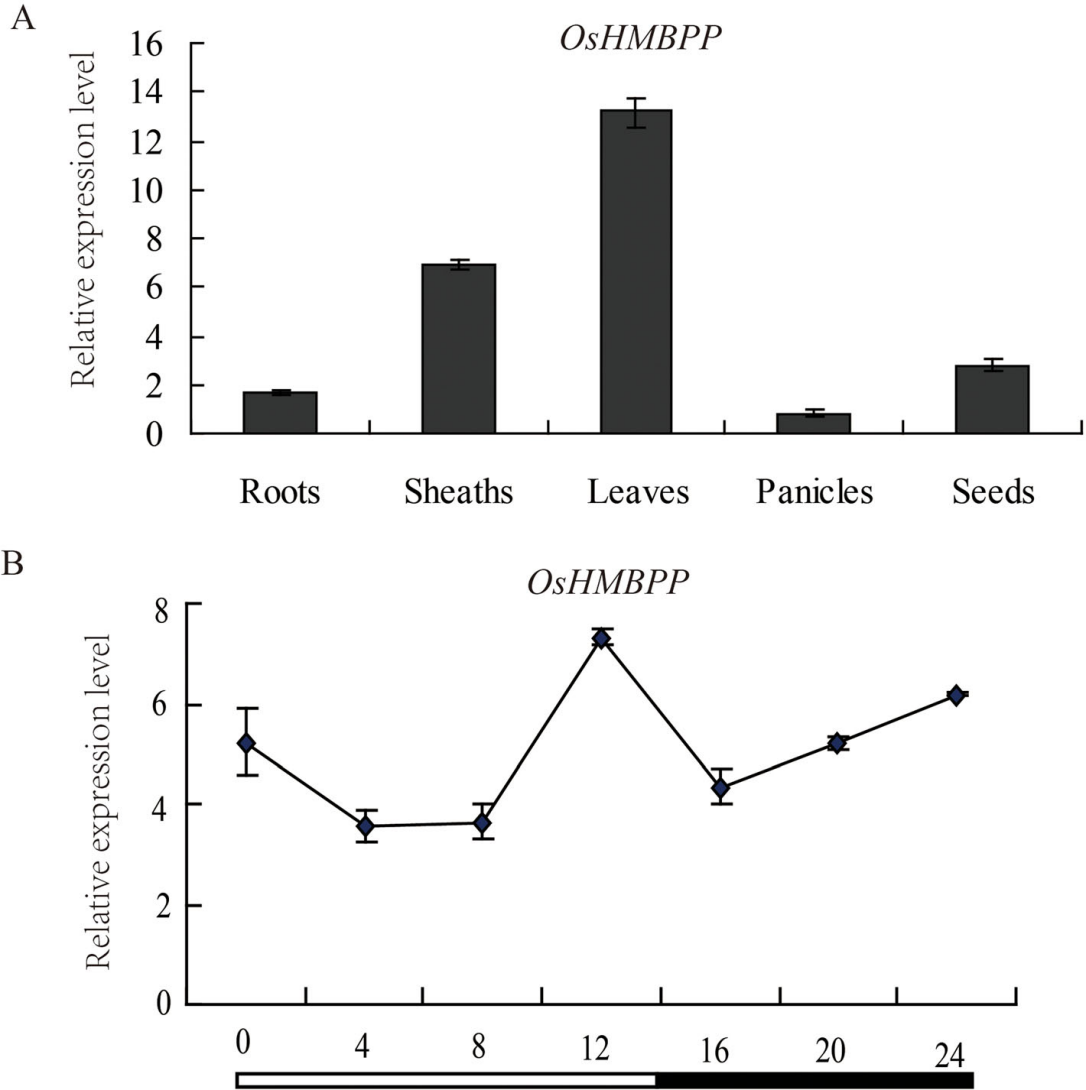
Expression analysis of *OsHMBPP*. **a** Expression of *OsHMBPP* in roots, sheaths, leaves, panicles, and seeds of wild-type plants. **b** Expression pattern of *OsHMBPP* in a 14-h light/ 10-h dark cycle. Values represent the mean ± SD of three biological replicates

According to the Rice eFP Browser (http://bar.utoronto.ca/efprice/cgi-bin/efpWeb.cgi), we found that *OsHMBPP* was expressed in all tissues, especially in leaves (Additional file 1: Figure S2). To verify these data, we examined the expression level of *LAS1* in different organs of the wild type by qRT-PCR. *OsHMBPP* was highly expressed in the mature leaves and also detected in the sheaths, seeds, roots and panicles (Fig. 3a). Our results demonstrated that *OsHMBPP* is constitutively expressed in various tissues and mainly functions in the green tissues.

To further investigate whether the expression of *OsHMBPP* is modulated by photoperiod, we compared the day/night expression patterns during the 14-h light/10-h dark photoperiod conditions. Test samples were taken every 4 h. qRT-PCR showed the expression of *OsHMBPP* oscillated during a light/dark cycle and the highest transcript level was detected during light period (Fig. 3b).

### *LAS1/OsHMBPP* Encodes a Conserved Chloroplast-Targeted Protein

**Fig. 4:**
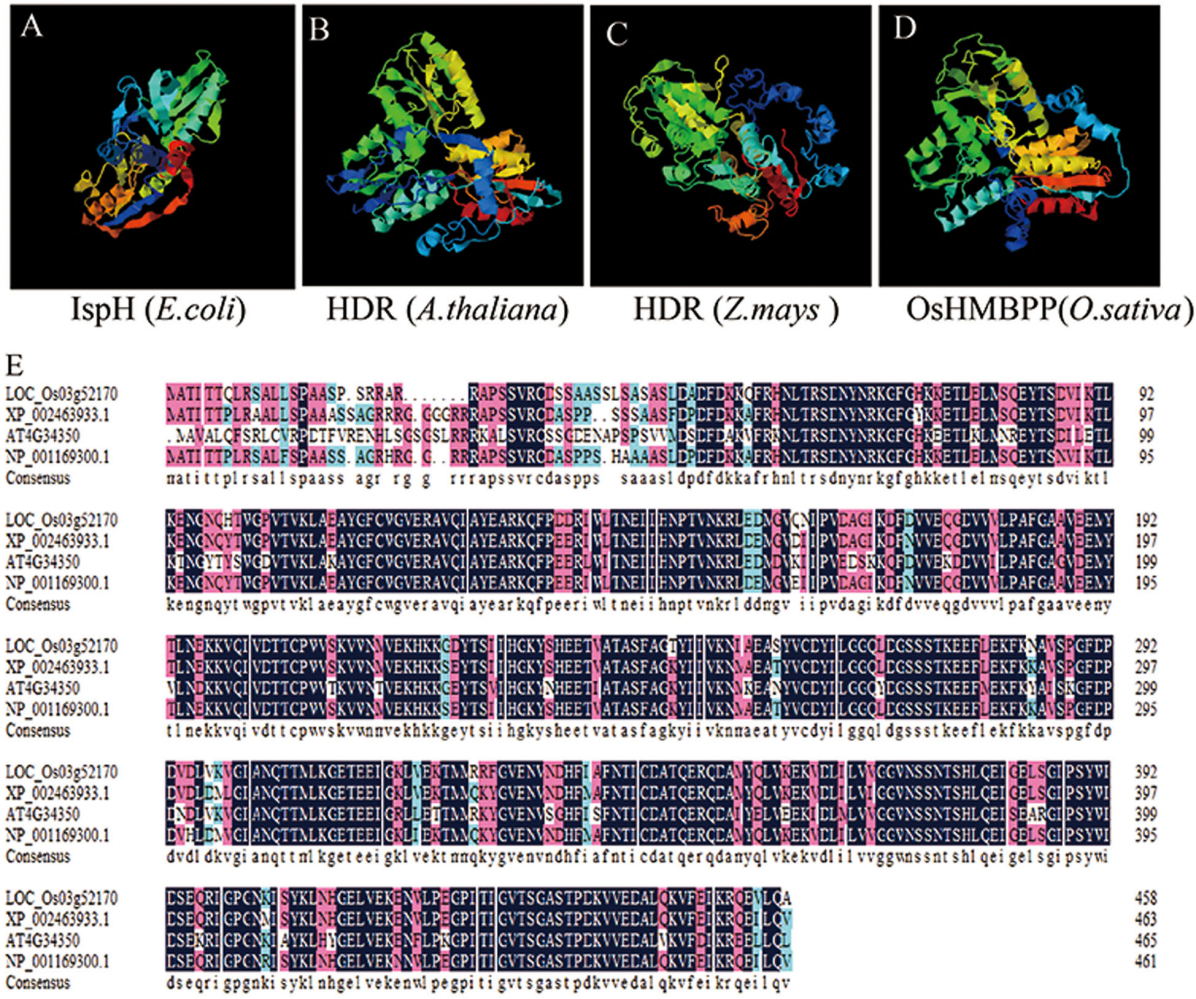
Protein structure of OsHMBPP and sequence alignment of multiple amino acids from different organisms. **a** Structure prediction of the HMBPP reductase IspH (*Escherichia coli*), HDR (*Arabidopsis*), HDR(*Zea mays*) and OsHMBPP (*Oryza sativa*) by I-TASSER algorithm. **b** Amino acid sequence alignment of the 3 types of OsHMBPP homologs. Amino acids that were fully or partially conserved are shaded blue and green, respectively

**Fig. 5:**
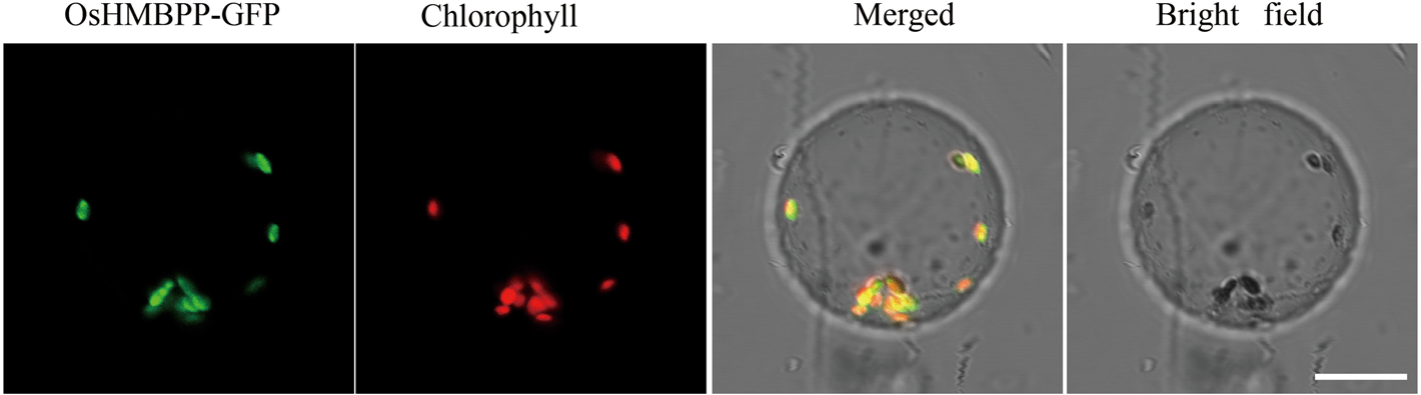
Subcellular location of the OsHMBPP protein. GFP signals of OsHMBPP-GFP fusion protein was located in the chloroplasts by transient expression analyses in rice protoplasts. Green fluorescence shows GFP, red fluorescence shows chloroplast autofluorescence, and yellow fluorescence shows the merged fluorescence

*LAS1/OsHMBPP* encodes a HMBPP reductase, which is involved in catalyzing 2C-methyl-D-erythritol4-phosphate (MEP) converted to IPP and DMAPP in the nonMVA pathway in *Arabidopsis thaliana* and maize, and affect the development of chloroplasts (Hsieh and Goodman, 2005; Lu et al. 2012). Therefore, we measured the contents of IPP and DMAPP between WT and *las1.* IPP and DMAPP contents in the *las1* seedlings were significantly reduced, indicating that the nonMVA pathway in *las1* was impaired (Additional file 1: Figure S3). Based on amino acid sequence alignment, OsHMBPP shares high identity with its orthologs in other species, including *Sorghum bicolor* (89.7%), *Arabidopsis* (73.8%), and *Zea mays* (90.1%) (Fig. 4b). Given that OsHMBPP and *Arabidopsis*, HDR, share 73.8% of amino acid identity and that structural predictions of both proteins revealed three-dimensional similarities by I-TASSER algorithm (Fig. 4a). ChloroP analysis revealed that the OsHMBPP protein contains a predicted chloroplast transit peptide of 31 amino acid residues at its N-terminal (Additional file 1: Table S1). To further determine its subcellular localization, a transient expression system was performed in rice protoplasts. As expected, the fusion protein OsHMBPP-GFP was clearly co-localized with Chl auto-fluorescence (Fig. 5), indicating OsHMBPP was localized in chloroplasts.

### Altered Expression of Chlorophyll Biosynthesis and Plastid-Encoded Genes

**Fig. 6:**
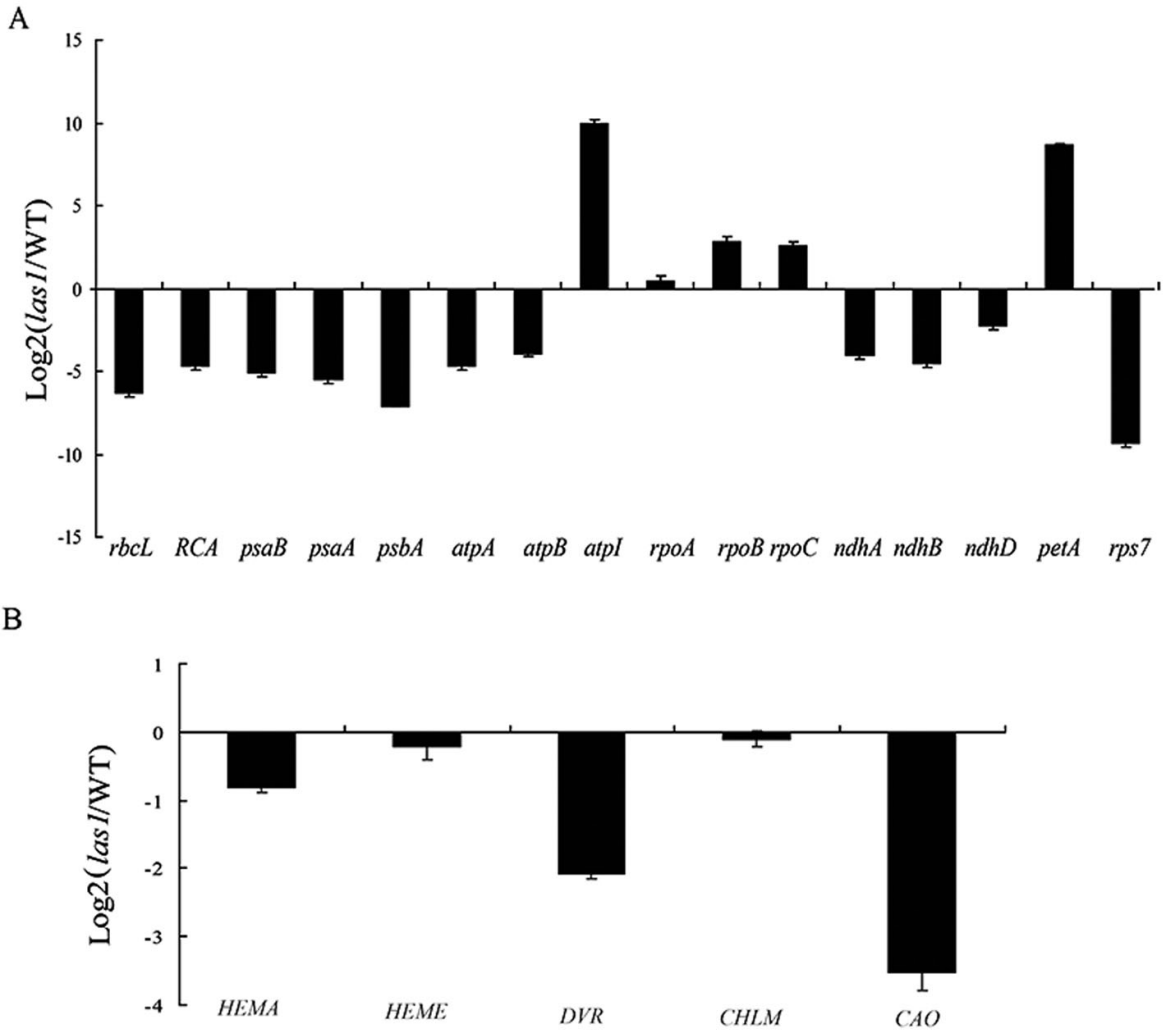
Expression analysis of genes involved in chloroplast development and chlorophyll biosynthesis. **a** Expression analysis of chloroplast development related genes in wild-type and *las1*. **b** Expression analysis of chlorophyll biosynthesis related genes in wild-type and *las1*. The relative expression level of each gene in WT plants was set to 1.0. *Ubiquitin* gene was used as a reference. *Error bars* indicate SD (*n* = 3)

We examined the expression levels of nuclear genes associated with Chl biosynthesis. qRT-PCR analyses showed that some Chl biosynthesis related genes were significantly down-regulated in the *las1* mutant (Fig. 6b). To investigate whether the *LAS1* mutation affects transcription by PEP and NEP, we examined the transcript abundance of various plastidic genes in the *las1* mutant by qRT-PCR. Plastid genes consist of three types of genes, which are transcribed by NEP, PEP, and both NEP and PEP, respectively (Yu et al. 2014). In *las1*, the expression levels of class III genes (e.g *rpoA*, *rpoB*, *rpoC*) were strongly increased, while class I genes (e.g *psaA*, *psbA*, *rbcL*) decreased (Fig. 6a). These results suggest that *las1* was defective in PEP activity and influenced the optimal expression of plastidic genes in rice seedlings.

### Mutation in *LAS1* Affects Four Plastdic Editing Sites and Chloroplast Ribosome Biogenesis

**Fig. 7:**
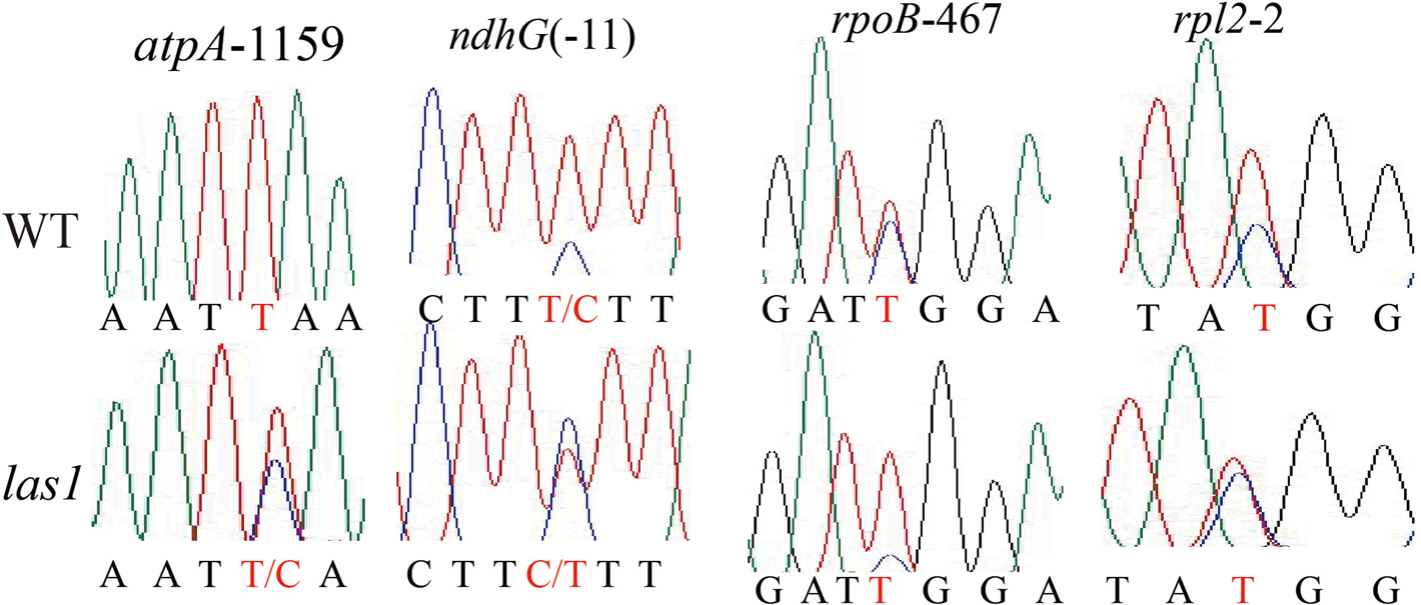
The OsHMBPP protein is required for RNA editing at multiple sites

The translation deficiency in *las1* suggests that *LAS1* might be involved in posttranscriptional RNA metabolism processes. We next detected whether *LAS1* was involved in the chloroplast RNA editing and RNA splicing. There are 21 chloroplast RNA editing sites in the 12 chloroplast genes (Corneille et al. 2000). Therefore, we analysed all 21 sites in the chloroplast cDNA in wild type and *las1*. We found that four C sites of chloroplast genes in *las1* did not edit normally. The editing of *rpl2-*C2, *ndhG-*C(− 11), and *atpA-*C1159 were reduced in *las1*, but the editing of *rpoB-*C467 was increased (Fig. 7). There was no difference between the wild type and *las1* in the rest 17 RNA editing sites (Additional file 1: Figure S4). There are 18 introns in the rice chloroplast genome (Kaminaka et al. 1999). Furthermore, we carried out RT-PCR with primers flanking the introns and compared the PCR products length between wild type and *las1* by agarose gel. Splicing of the 17 chloroplast transcripts were not significantly impeded in *las1* (Additional file 1: Figure S5**)**. These data suggest that *LAS1* affects the RNA editing efficiency of chloroplast genes, but not function in chloroplast RNA splicing.

**Fig. 8:**
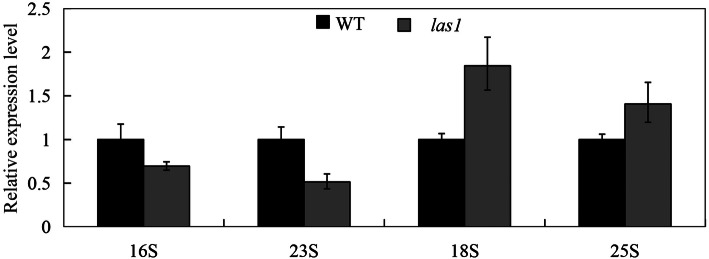
The expression level of 16 S, 23 S, 18 S and 25 S in wild type and *las1*. *Error bars* represent the SD from three independent experiments

Abnormal chloroplast development is often associated with inhibition of chloroplast ribosome synthesis (Ge et al. 2017; Cao et al. 2019), which is composed of 30 S small subunit and 50 S large subunit. We further examined whether the activity of chloroplast ribosome changed in *las1*. qRT-PCR analysis indicated that the expression levels of *16 S* and *23 S* were decreased whereas *18 S* and *25 S* were increased in *las1*, compared with the wild type (Fig. 8). The result indicated that *las1* was defective in chloroplast ribosome biogenesis.

### LAS1/OsHMBPP Interacts with the MORF Family Protein in Yeasts

**Fig. 9:**
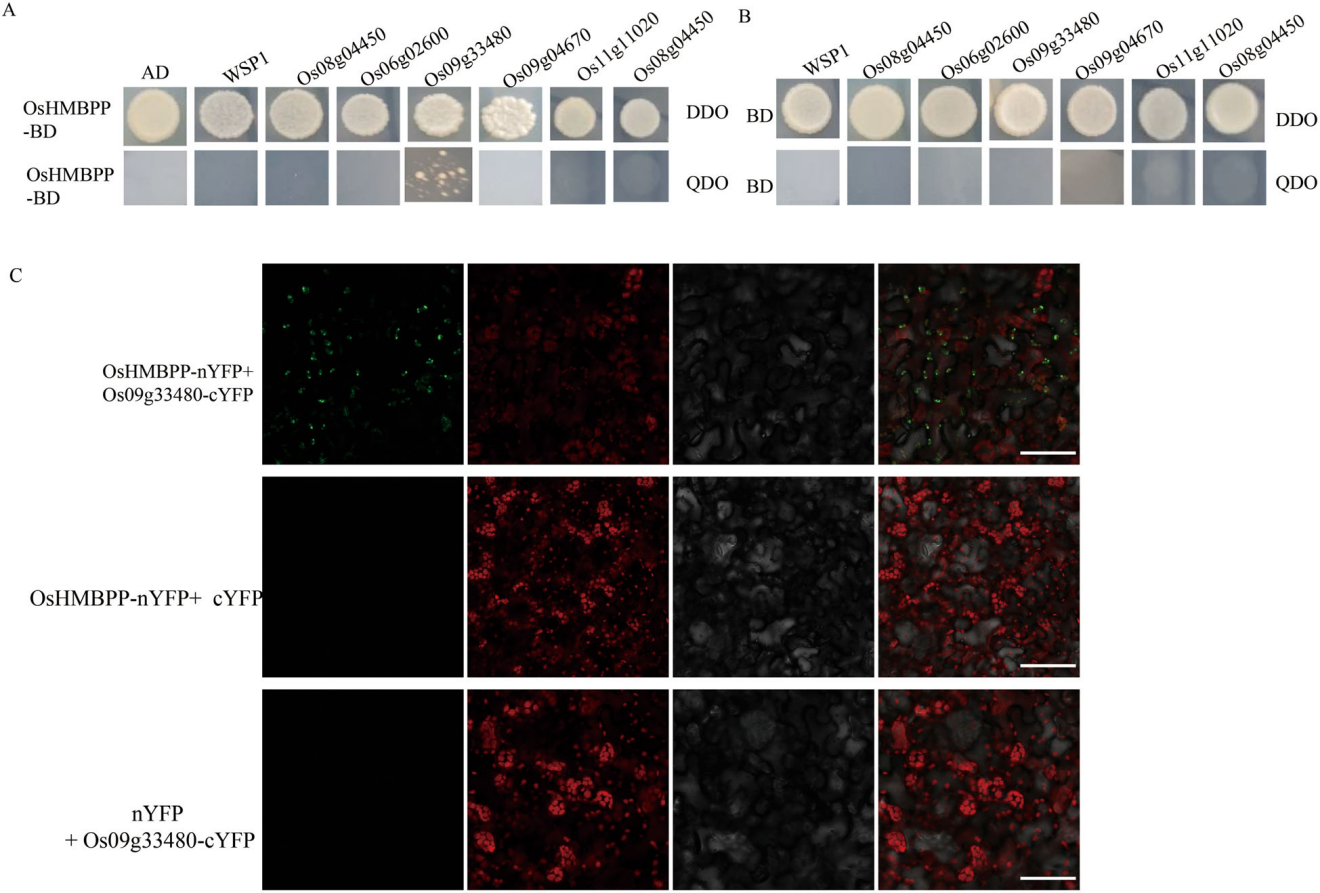
The interaction assay between OsHMBPP and MORF families in rice. **a**-**b***OsHMBPP* was fused to the pGBKT7 vector (OsHMBPP-BD). Seven MORFs were fused to the pGADT7 vector. DDO, control medium (SD–Leu/−Trp); QDO, selective medium (SD–Leu/−Trp/−His/−Ade). **c** Interaction between OsHMBPP and Os09g33480 in BiFC assays. Bars = 100 μm

The multiple organellar RNA editing factor (MORF) family proteins are essential for the chloroplast/mitochondrion RNA editing in plants (Bentolila et al. 2012; Takenaka et al. 2012; Zhang et al. 2017). There are nine MORF proteins and seven MORF proteins in *Arabidopsis* and rice, respectively. To investigate whether LAS1/OsHMBPP interacted with the MORF family proteins, we used the yeast two hybrid to detect the interaction. Notably, the results showed that only Os09g33480 had an interaction with OsHMBPP (Fig. 9a-b). Furthermore, we performed BiFC assays using *N. benthamiana* cells and found that OsHMBPP-nYFP and Os09g33480-cYFP could interact with each other (Fig. 9c). Os09g33480 belongs to the *Arabidopsis* MORF8 branch. Os09g33480 may be targeted to both mitochondria and chloroplasts similar to *Arabidopsis* MORF8. Meanwhile, WSP1, Os06g02600, Os09g04670, Os08g04450, Os11g11020, and Os03g38490 did not interact with OsHMBPP (Fig. 9a-b). Based on the Rice eFP Browser, *Os09g33480* is ubiquitously expressed in many rice organs, especially in seeds, panicles and leaves (Additional file 1: Figure S6).

### Expression Patterns of the MEP Pathway Genes in a Normal Day/Night Cycle

To further investigate whether expression of the MEP pathway genes is regulated by photoperiod, we compared the expression patterns of six genes during the 14-h light/10-h dark photoperiod conditions (Additional file 1: Figure S7). The expression patterns of *CMK* and *DXS* were similar during the 14-h-light/10-h-dark cycle. Peak levels of *CMK* and *DXS* were detected in the early period of the light cycle (8–12 h in the light). Oscillations in the expression of *DXR*, *HDS*, *MCS*, and *CMS* also occurred during the light/dark cycle. In contrast to *CMK* and *DXS*, peak levels of *DXR* and *HDS* expression appeared in the late period of the dark cycle (20-24 h). The expression of all MEP pathway genes is significantly repressed during the transition from light to dark (Additional file 1: Figure S7).

## Discussion

### Characterization of *LAS1/OsHMBPP*

Chloroplast defective development generally results in an albino or chlorotic leaf phenotype, which severely affects the biomass and even the survival of plants, particularly in crops. Isoprenoids play an essential role in chloroplast development. In this study, we isolated the *las1* mutant, which displayed an albino leaf phenotype and was seedling lethal (Fig. 1). TEM observation demonstrated that the phenotype of *las1* resulted from the defective development of thylakoids, eventually leading to the breakdown of entire chloroplasts (Fig. 2). *LAS1* encodes 4-hydroxy-3-methylbut-2-enyl diphosphate reductase OsHMBPP in rice (Fig. 5).HMBPP functions in the isoprenoid biosynthesis via the MEP pathway (McAteer et al. 2001; Guevara-Garcia et al. 2005; Hsieh and Goodman, 2005). Isoprenoids are derived from a basic five-carbon unit, isopentenyl diphosphate (IPP) and allyl isomerdimethylallyl diphosphate (DMAPP). HMBPP is the rate limiting enzyme of this process, and regulates the biosynthesis of chlorophylls, ABA and GA. Mutation of HMBPP in maize and *A. thaliana* produced an albino phenotype with impaired chloroplasts containing large vesicles (Hsieh and Goodman, 2005; Lu et al. 2012). In rice, the *OsHMBPP* mutation resulted in a reduction in the content of IPP and DMAPP (Additional file 1: Figure S3), which led to decrease of the chlorophyll content and plant death. On the other hand, OsHMBPP interacted with the MORF protein Os09g33480(Fig. 9), and affected the RNA editing of chloroplast genes. These results indicate that the function of HMBPP is very conserved in different species, and is essential for the early chloroplast development in rice.

### *LAS1* Encodes a Chloroplast-Targeted Protein and Regulates the Early Chloroplast Development in Rice

Our results showed that *LAS1* was constitutively expressed, with high levels of transcript in the leaves, implying that *LAS1* might play an essential role in leaf development (Fig. 3a). Subcellular localization in rice protoplasts demonstrated that the LAS1 protein is routed to the chloroplasts (Fig. 4). The chloroplast is a semi-autonomous organelle, which contains about 100 genes, although more than 3000 proteins function within it (Leister, 2003). Thus, nucleus-encoded factors play essential roles in regulating chloroplast development, which requires the coordinated expression of both nucleus-encoded and chloroplast-encoded genes. The *LAS1* mutation disrupts the transcripts of plastid and nuclear genes associated with chloroplast development (Fig. 6). The transcript accumulation of both PEP-and NEP-dependent genes(*atpB*) and PEP-transcribed plastid genes (*psaA, psbA, rbcL*) were severely suppressed (Fig. 6), suggesting that the accumulation of transcripts for PEP components did not result in the formation of functional PEP due to the disruption of transcription/ translation apparatus.

### *OsHMBPP* Is Required for Chloroplast Genes Transcript Editing

RNA editing is an important event in post-transcriptional RNA process, which alters RNA sequences by converting specific target cytidines to uridine in transcripts of plastids and mitochondria (Takenaka et al. 2013). Abolishing editing in plastidic genes can disturb plastidic functionality and inhibit rice normal development and growth (Tang et al. 2017; Zhang et al. 2017; Liu et al. 2018; Cui et al. 2019). For example, in the *wsp1* mutant, editing at *ndhD*-878, *rps14*–80, *ndhG*(− 11), and *rpoB*-467, 545, 560 are abolished, resulting in the defective chloroplast development. Meanwhile, the mutation of pentatricopeptide repeat protein gene *DUA1* reduced the editing efficiency of *rps*-182 and *rpoB*-545, 560, leading to the seedling lethality under cold stress. Our findings showed that the editing efficiency of four chloroplast RNA editing sites in *las1* is remarkably altered, compared with the wild type (Fig. 7), which may be an explanation for the phenotype of *las1*. Edited *rpoB* codon 467 is located in the Dispensable Region I and the editing of the *rpoB*-467 site is non-essential (Corneille et al. 2000). In rice, the *rpoB*-467 site is partial editing. Therefore, the altered editing of the *rpoB*-467 site was not the cause of the phenotype of *las1*. The chloroplast and mitochondria RNA editing events may be an indication of evolutionary adaptation (Chu and Wei, 2019). The *rpl2*, *rpoB* and *atpA* gene sequences of the chloroplast genomes of different species were analyzed from the perspective of evolution. The *rpoB*-467 and *atpA*-1159 encode a Ser in monocots, but encode either a Ser or a Leu in eudicots (Additional file 1: Figure S8). We found an interesting phenomenon that *rpl2*–2 encodes Thr in monocots, but it encodes Met in eudicots (Additional file 1: Figure S8). These results showed that the *rpl2*–2 sites of monocots and eudicots were highly differentiated. The ‘C’ at *rpl2*–2 of monocots may have been acquired during the evolution.

Plant MORF proteins are essential for RNA editing and splicing, but they are rarely reported in rice. Recently, A MORF2-like protein, WSP1, was identified as a rice RNA editing/splicing factor (Zhang et al. 2017). The *WSP1* mutation affects the RNA editing of *ndhG* and *rpoB*, suggesting that WSP1 and OsHMBPP might cooperate together for RNA editing. Therefore, we tested the interaction between OsHMBPP, WSP1 and other six MORF proteins. Y2H assays showed that OsHMBPP and WSP1 did not interact together in vivo (Fig. 9). However, we confirmed that OsHMBPP can interact with Os09g33480 by Y2H and BIFC assays (Fig. 9a-c), but not interact with the other six MORFs in yeasts (Fig. 9a). Our results suggested that *OsHMBPP* may act as an organellar RNA editing factor via an editosome coupled with Os09g33480.

## Conclusions

We isolated a novel seedling-lethal albino rice mutant *las1* by the CRISPR/Cas9 system. Mutation in the HMBPP reductase is responsible for the phenotype of *las1*. Our results demonstrate that *LAS1/OsHMBPP* is important for plastid gene expression and normal chloroplast development. Also, we found the plastid RNA editing in *las1* was altered, and LAS1/OsHMBPP interacted with the MORF protein Os09g33480 in vivo. Further studies on identifying the mutant phenotype of other MEP pathway genes, and how the rice MEP pathway enzymes are regulated.

## Methods

### Plant Materials and Growth Conditions

The *las1* mutant was obtained from the pool of the *japonica* cultivar Nipponbare by the CRISPR/Cas9 system. The CRISPR/Cas9 binary vectors were constructed as previously described (Lu et al. 2017). The seedlings were grown in a growth chamber or in the field. The growth chamber condition was set as follow:14-h light at 30 °C and 10-h dark at 25 °C.

### Measurement of Pigment Contents

Pigments were extracted from fresh leaf samples (0.2 g) from wild-type and las1 plants that were cut into small segments and incubated with 5 ml of 95% ethanol in the dark for 48 h. The supernatant was measured with a dual-beam ultraviolet spectrophotometer (TU1901, Beijing) at 470, 645, and 663 nm. Chl a, Chl b, and carotenoid (Car) contents were measured according to the methods by Porra et al. (1989). Three biological replicates were analyzed.

### Transmission Electron Microscopy (TEM)

For TEM analysis, transverse sections of leaf samples were taken from *las1* and WT seedlings. Leaf samples were fixed in 2.5% glutaraldehyde and then in 1% OsO_4_. Samples were prepared as described previously (Gothandam et al. 2005), and were observed using a Jeol 1230 electron microscope (Tokyo, Japan).

### Protein Structure and Sequence Alignment Analysis

The predicted full-length LAS1 protein sequence, with 459 amino acids, was obtained from RGAP (http://rice.plantbiology.msu.edu/). The sequences used in the alignment analysis were obtained by a BLASTP search using the LAS1 protein sequence as the query at the National Center for Biotechnology Information (NCBI, http://www.ncbi.nlm.nih.gov/). The full-length amino acid sequences were aligned using the DNAMAN.

### Measurement of IPP and DMAPP Contents

IPP and DMAPP contents were determined by the specific ELISA Kit from the Sci-tech innovation company (Qingdao, China). Baed on the manufacturer’s instructions, each sample was assayed five times.

### Subcellular Localization

To investigate the subcellular localization of LAS1, we amplified the full-length cDNA of *LAS1* without the termination codon (Additional file 1: Table S2). The fragment was cloned into the GFP vector pAN580. The LAS1-GFP vector was transformed into rice protoplasts (Wang et al. 2016), and the transformed protoplasts were observed with a Zeiss LSM700 laser scanning confocal microscope.

### RNA Extraction and qRT-PCR Analysis

Total RNA was extracted and purified from different tissues using the RNApure Plant Kit (CWBIO) according to the manufacturer’s instructions. First strand cDNAs were synthesized from 2 μg total RNA using a PrimeScript 1st Strand cDNA Synthesis Kit (Takara). qRT-PCR was performed with a CFX96 Touch Real-time PCR Detection System using the SYBR Premix Ex TaqTM kit (Takara). The rice *UBQ* gene was used as an internal control. All primers for qRT-PCR are obtained as reported previously (Wang et al. 2016; Ge et al. 2017; Liu et al. 2018). The data were expressed as the mean ± SD of three biological replicates.

### RNA Editing Analysis

All 21 known rice chloroplast RNA editing sites were assayed as reported (Corneille et al. 2000). Specific cDNA fragments with the editing sites were amplified between wild type and *las1*, and then sequenced. The cDNA sequences were compared to identify C to T changes resulting from RNA editing by DNAMAN software. All primers for RNA editing analysis are obtained as reported previously (Wang et al. 2017).

### Yeast Two-Hybrid and Transient Expression Assays

Full-length coding sequences of *LAS1* was cloned into pGBKT7 vector, and seven MORF genes (*Os06g02600*, *WSP1*, *Os03g38490*, *Os09g33480*, *Os09g04670*, *Os11g11020*, *Os08g04450*) were cloned into pGADT7 vector, resulting in LAS1-BD and MORFs-AD respectively (Additional file 1: Table S2). The assay was performed following the manufacturer’s instructions (Clontech).

For Bimolecular Fluorescent Complimentary assay, OsHMBPP-nYFP, Os09g33480-cYFP and corresponding empty vectors were transformed into Agrobacterium strain EHA105 and transfected leaves of *N. benthamiana* (Additional file 1: Table S2). The fluorescence was observed with a confocal scanning microscopy (ZEISS).

## Supplementary information

**Additional file 1: Figure S1.** The phenotype and sequence confirmation of *las1*. **Figure S2.** Expression pattern of *OsHMBPP* at different growth stages. Data was collected from the Rice eFP Browser. **Figure S3.** Determination of IPP and DMAPP between WT and *las1*. **Figure S4.** The rest 17 chloroplast RNA editing sites between WT and *las1*. **Figure S5.** Splicing Analysis of rice chloroplast genes in wild-type and *las1*. **Figure S6.** Expression pattern of *Os09g33480* at different growth stages. Data was collected from the Rice eFP Browser. **Figure S7.** Expression patterns of rice nonMVA pathway genes in a 14 h light/ 10 h dark. Wild-type seedlings were grown in a growth chamber for 14 days after germination, and then the seedlings were sampled every 4 h. *Error bars* indicate SD (*n* = 3). **Figure S8.** RNA-edited amino acids alignment of the plastid rpl2, atpA and rpoB in monocot(1–6) and eudicot(7–12) . 1: *Zea mays*, 2: *Oryza sativa*, 3: *Hordeum vulgare*, 4: *Sorghum bicolor*, 5:Triticum aestivum, 6: *Phoenix dactylifera*, 7: *Arabidopsis thaliana*, 8:Brassica napus, 9: *Nicotiana tabacum*, 10: *Glycine max*, 11: *Vitis vinifera*, 12: *Gossypium arboreum*. **Table S1.** Chloroplast signal peptide prediction. **Table S2.** Primers used in real-time PCR and vector construction.

## Data Availability

All data supporting the conclusions of this article are provided within the article (and its additional files).

## Abbreviations

MVA: Mevalonate
MEP: Methyl-D-erythritol-4-phosphate
MORF: Multiple organellar RNA editing factor
Chl: Chlorophyll
rpl2: Ribosomal protein L2
TEM: Transmission electron microscopy
qRT-PCR: Quantitative real-time PCR
atpA: ATP synthase CF1 alpha subunit
PEP: Plastid-encoded RNA polymerase
NEP: Nuclear-encoded polymerase

## References

[CR1] Adam P, Hecht S, Eisenreich W, Kaiser J, Grawert T, Arigoni D, Bacher A, Rohdich F (2002). Biosynthesis of terpenes: studies on 1-hydroxy-2-methyl-2-(E)-butenyl 4-diphosphate reductase. Proc Natl Acad Sci U S A.

[CR2] Albrecht V, Ingenfel A, Apel K (2008). Snowy cotyledon 2: the identification of a zinc finger domain protein essential for chloroplast development in cotyledons but not in true leaves. Plant Mol Biol.

[CR3] Bentolila S, Heller WP, Sun T, Babina AM, Friso G, Wijk KJ, Hanson MR (2012). RIP1, a Member of an Arabidopsis Protein Family, Interacts With the Protein RARE1 and Broadly Affects RNA Editing. Proc Natl Acad Sci USA.

[CR4] Budziszewski GJ, Lewis SP, Glover LW, Reineke J, Jones G, Ziemnik LS, Lonowski J, Nyfeler B, Aux G, Zhou Q, McElver J, Patton DA, Martienssen R, Grossniklaus U, Ma H, Law M, Levin JZ (2001). *Arabidopsis* genes essential for seedling viability: isolation of insertional mutants and molecular cloning. Genetics.

[CR5] Cao P, Ren Y, Liu X, Zhang T, Zhang P, Xiao L, Zhang F, Liu S, Jiang L, Wan JM (2019). Purine nucleotide biosynthetic gene *GARS* controls early chloroplast development in rice (*Oryza sativa* L.). Plant Cell Rep.

[CR6] Chu D, Wei L (2019). The chloroplast and mitochondrial C-to-U RNA editing in *Arabidopsis thaliana* shows signals of adaptation. Plant Direct.

[CR7] Corneille S, Lutz K, Maliga P (2000). Conservation of RNA editing between rice and maize plastids: are most editing events dispensable?. Mol Gen Genet.

[CR8] Cui X, Wang Y, Wu J, Han X, Gu X, Lu T, Zhang Z (2019). The RNA editing factor *DUA1* is crucial to chloroplast development at low temperature in rice. New Phytol.

[CR9] Cunningham FX, Lafond TP, Gantt E (2000). Evidence of a role for LytB in the nonmevalonate pathway of isoprenoid biosynthesis. J Bacteriol.

[CR10] Disch A, Hemmerlin A, Bach T, Rohmer M (1998). Mevalonate-derived isopentenyl diphosphate is the biosynthetic precursor of ubiquinone prenyl side chain in tobacco BY-2 cells. J Biochem.

[CR11] Eisenreich W, Schwarz M, Cartayrade A, Arigoni D, Zenk MH, Bacher A (1998). The deoxyxylulose phosphate pathway of terpenoid biosynthesis in plants and microorganisms. Chem Biol.

[CR12] Ge C, Wang L, Ye W, Wu L, Cui Y, Chen P, Pan J, Zhang D, Hu J, Zeng D, Dong G, Qian Q, Guo L, Xue D (2017). Single-point mutation of an histidine-aspartic domain-containing gene involving in chloroplast ribosome biogenesis leads to white fine stripe leaf in rice. Sci Rep.

[CR13] Gothandam KM, Kim ES, Cho H, Chung YY (2005). OsPPR1, a pentatricopeptide repeat protein of rice is essential for the chloroplast biogenesis. Plant Mol Biol.

[CR14] Guevara-Garcia A, San Roman C, Arroyo A, Cortes ME, de la Luz G-NM, Leon P (2005). Characterization of the *Arabidopsis clb6* mutant illustrates the importance of posttranscriptional regulation of the methyl-D-erythritol 4-phosphate pathway. Plant Cell.

[CR15] Gutierrez-Nava ML, Gillmor CS, Jimenez LF, Guevara-Garcia A, Leon P (2004). *CHLOROPLAST BIOGENESIS* genes act cell and noncell autonomously in early chloroplast development. Plant Physiol.

[CR16] Hecht S, Eisenreich W, Adam P, Amslinger S, Kis K, Bacher A, Arigoni D, Rohdich F (2001). Studies on the nonmevalonate pathway to terpenes: the role of the GcpE (IspG) protein. Proc Natl Acad Sci U S A.

[CR17] Herz S, Wungsintaweekul J, Schuhr CA, Hecht S, Luttgen H, Sagner S, Fellermeier M, Eisenreich W, Zenk MH, Bacher A, Rohdich F (2000). Biosynthesis of terpenoids: YgbB protein converts 4-diphosphocytidyl-2C-methyl-D-erythritol 2-phosphate to 2C-methyl-D-erythritol 2,4- cyclodiphosphate. Proc Natl Acad Sci U S A.

[CR18] Hsieh MH, Goodman HM (2005). The Arabidopsis IspH homolog is involved in the plastid non mevalonate pathway of isoprenoid biosynthesis. Plant Physiol.

[CR19] Jarvis P, López-Juez E (2013). Biogenesis and homeostasis of chloroplasts and other plastids. Nat Rev Mol Cell Biol.

[CR20] Kaminaka H, Morita S, Tokumoto M, Yokoyama H, Masumura T, Tanaka K (1999). Molecular cloning and characterization of a cDNA for an iron-superoxide dismutase in rice (*Oryza sativa* L.). Biosci Biotechnol Biochem.

[CR21] Kessler F, Schnell D (2009). Chloroplast biogenesis: diversity and regulation of the protein import apparatus. Curr Opin Cell Biol.

[CR22] Leister D (2003). Chloroplast research in the genomic age. Trends Genet.

[CR23] Lichtenthaler HK (1999). The 1-deoxy-D-xylulose-5-phosphate pathway of isoprenoid biosynthesis in plants. Annu Rev Plant Biol.

[CR24] Liu X, Lan J, Huang YS, Cao PH, Zhou CL, Ren YK, He NQ, Liu SJ, Tian YL, Nguyen T, Jiang L, Wan JM (2018). WSL5, a pentatricopeptide repeat protein, is essential for chloroplast biogenesis in rice under cold stress. J Exp Bot.

[CR25] Liu X, Rodermel SR, Yu F (2010). A *var2* leaf variegation SUPPRESSOR locus, *SUPPRESSOR OF VARIEGATION3*, encodes a putative chloroplast translation elongation factor that is important for chloroplast development in the cold. BMC Plant Biol.

[CR26] Lois LM, Campos N, Putra SR, Danielsen K, Rohmer M, Boronat A (1998). Cloning and characterization of a gene from *Escherichia coli* encoding a transketolase-like enzyme that catalyzes the synthesis of D-1-deoxyxylulose 5-phosphate, a common precursor for isoprenoid, thiamin, and pyridoxol biosynthesis. Proc Natl Acad Sci U S A.

[CR27] Lopez-Juez E, Pyke KA (2005). Plastids unleashed: their development and their integration in plant development. Int J Dev Bio.

[CR28] Lu XM, Hu XJ, Zhao YZ, Song WB, Zhang M, Chen ZL, Chen W, Dong YB, Wang ZH, Lai JS (2012). Map-based cloning of *zb7* encoding an IPP and DMAPP synthase in the MEP pathway of maize. Mol Plant.

[CR29] Lu Y, Ye X, Guo R, Huang J, Wang W, Tang JY, Tan LT, Zhu JK, Chu CC, Qian YW (2017). Genome-wide targeted mutagenesis in rice using the CRISPR/Cas9 system. Mol Plant.

[CR30] McAteer S, Coulson A, McLennan N, Masters M (2001). The *lytB* gene of Escherichia coli is essential and specifies a product needed for isoprenoid biosynthesis. J Bacteriol.

[CR31] Page JE, Hause G, Raschke M, Gao W, Schmidt J, Zenk MH, Kutchan TM (2004). Functional analysis of the final steps of the 1-deoxy-D-xylulose 5-phosphate (DXP) pathway to isoprenoids in plants using virusinduced gene silencing. Plant Physiol.

[CR32] Pogson BJ, Albrecht V (2011). Genetic dissection of chloroplast biogenesis and development: an overview. Plant Physiol.

[CR33] Porra RJ, Thompson WA, Kriedemann PE (1989). Determination of accurate extinction coefficients and simultaneous equations for assaying chlorophylls a and b extracted with four different solvents: verification of the concentration of chlorophyll standards by atomic absorption spectroscopy. BBA-Bioenergetics.

[CR34] Rodriguez-Concepcion M, Boronat A (2002). Elucidation of the methylerythritol phosphate pathway for isoprenoid biosynthesis in bacteria and plastids. A metabolic milestone achieved through genomics. Plant Physiol.

[CR35] Rohmer M (2003). Mevalonate-independent methylerythritol phosphate pathway for isoprenoid biosynthesis: elucidation and distribution. Pure Appl Chem.

[CR36] Rohmer M, Knani M, Simonin P, Sutter B, Sahm H (1993). Isoprenoid biosynthesis in bacteria: a novel pathway for the early steps leading to isopentenyl diphosphate. Biochem J.

[CR37] Takenaka M, Zehrmann A, Verbitskiy D, Kugelmann M, Hartel B, Brennicke A (2012). Multiple organellar RNA editing factor (MORF) family proteins are required for RNA editing in mitochondria and plastids of plants. Proc Natl Acad Sci USA.

[CR38] Takenaka M, Zehrmann A, Verbitskiy D, Hartel B, Brennicke A (2013). RNA editing in plants and its evolution. Annu Rev Genet.

[CR39] Tang JP, Zhang WW, Wen K, Chen GM, Sun J, Tian YL, Tang WJ, Yu J, An HZ, Wu TT, Kong F, Terzaghi W, Wang CM, Wan JM (2017). OsPPR6, a pentatricopeptide repeat protein involved in editing and splicing chloroplast RNA, is required for chloroplast biogenesis in rice. Plant Mol Biol.

[CR40] Wang P, Grimmm B (2015). Organization of chlorophyll biosynthesis and insertion of chlorophyll into the chlorophyll-binding proteins in chloroplasts. Photosynth Res.

[CR41] Wang Y, Ren Y, Zhou K, Liu L, Wang J, Xu Y, Zhang H, Zhang L, Feng Z, Wang L, Ma W, Wang Y, Guo X, Zhang X, Lei C, Cheng Z, Wan JM (2017). *WHITE STRIPE LEAF4* encodes a novel P-type PPR protein required for chloroplast biogenesis during early leaf development. Front Plant Sci.

[CR42] Wang Y, Wang C, Zheng M, Lyu J, Xu Y, Li X, Niu M, Long W, Wang D, Wang H, Terzaghi W, Wang Y, Wan JM (2016). *WHITE PANICLE1*, a val-tRNA synthestase regulating chloroplast ribosome biogenesis in rice, is essential for early chloroplast development. Plant Physiol.

[CR43] Yu QB, Huang C, Yang ZN (2014). Nuclear-encoded factors associated with the chloroplast transcription machinery of higher plants. Front Plant Sci.

[CR44] Zagari N, Sandoval-Ibañez O, Sandal N, Su J, Rodriguez-Concepcion M, Stougaard J, Pribil M, Leister D, Pulido P (2017). *SNOWY COTYLEDON 2* promotes chloroplast development and has a role in leaf variegation in both *lotus japonicus* and *Arabidopsis thaliana*. Mol Plant.

[CR45] Zeidler J, Lichtenthaler H, May H, Lichtenthaler F (1997). Is isoprene emitted by plants synthesized via the novel isopentenyl pyrophosphate pathway?. Z Naturforsch C.

[CR46] Zhang Z, Cui X, Wang Y, Wu J, Gu X, Lu T (2017). The RNA editing factor *WSP1* is essential for chloroplast development in rice. Mol Plant.

