# Supplementary material for: Disruption of a Rice Chloroplast-Targeted Gene OsHMBPP Causes a Seedling-Lethal Albino Phenotype
Source: Rice (N Y). 2020 Jul 25;13:51. doi: 10.1186/s12284-020-00408-1 (PMC7382669; doi:10.1186/s12284-020-00408-1)
Supplement: Supplementary file 1 — Additional file 1: Figure S1. The phenotype and sequence confirmation of las1. Figure S2. Expression pattern of OsHMBPP at different growth stages. Data was collected from the Rice eFP Browser. Figure S3. Determination of IPP and DMAPP between WT and las1. Figure S4. The rest 17 chloroplast RNA editing sites between WT and las1. Figure S5. Splicing Analysis of rice chloroplast genes in wild-type and las1. Figure S6. Expression pattern of Os09g33480 at different growth stages. Data was collected from the Rice eFP Browser. Figure S7. Expression patterns of rice nonMVA pathway genes in a 14 h light/ 10 h dark. Wild-type seedlings were grown in a growth chamber for 14 days after germination, and then the seedlings were sampled every 4 h. Error bars indicate SD (n = 3). Figure S8. RNA-edited amino acids alignment of the plastid rpl2, atpA and rpoB in monocot(1–6) and eudicot(7–12) . 1: Zea mays, 2: Oryza sativa, 3: Hordeum vulgare, 4: Sorghum bicolor, 5:Triticum aestivum, 6: Phoenix dactylifera, 7: Arabidopsis thaliana, 8:Brassica napus, 9: Nicotiana tabacum, 10: Glycine max, 11: Vitis vinifera, 12: Gossypium arboreum. Table S1. Chloroplast signal peptide prediction. Table S2. Primers used in real-time PCR and vector construction. [file 12284_2020_408_MOESM1_ESM.doc]

**RUNNING TITLE:** *LAS1/OsHMBPP* regulates chloroplast development in rice

**Xi Liu**
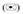
 **•Penghui Cao • Qianqian Huang** **•Yanrong Yang• Dandan Tao**

Disruption of a rice chloroplast-targeted gene *OsHMBPP* causes a seedling-lethal albino phenotype

**X. Liu^1^**
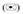
* **• P.H. Cao^2^*** **• Q.Q. Huang^1^•Y.R. Yang^1^ • D.D. Tao^1^**

1 Key Laboratory of Eco-Agricultural Biotechnology around Hongze Lake, Regional Cooperative Innovation Center for Modern Agriculture and Environmental Protection, Huaiyin Normal University, Huaian 223300, China.

2 Suzhou Academy of Agricultural Sciences, Suzhou 215155, China.

Corresponding author: Xi Liu, Associate professor

Telephone: +86-15195985198

E-mail: 1240623244@qq.com

*These authors contributed equally to this work.

**
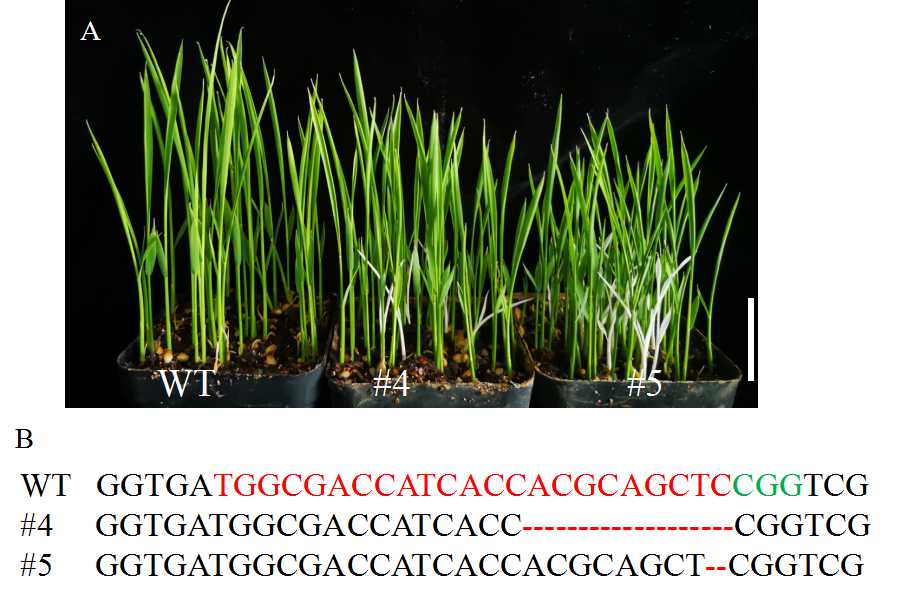
**

Supplemental Figure 1. The phenotype and sequence confirmation of *las1*.

**
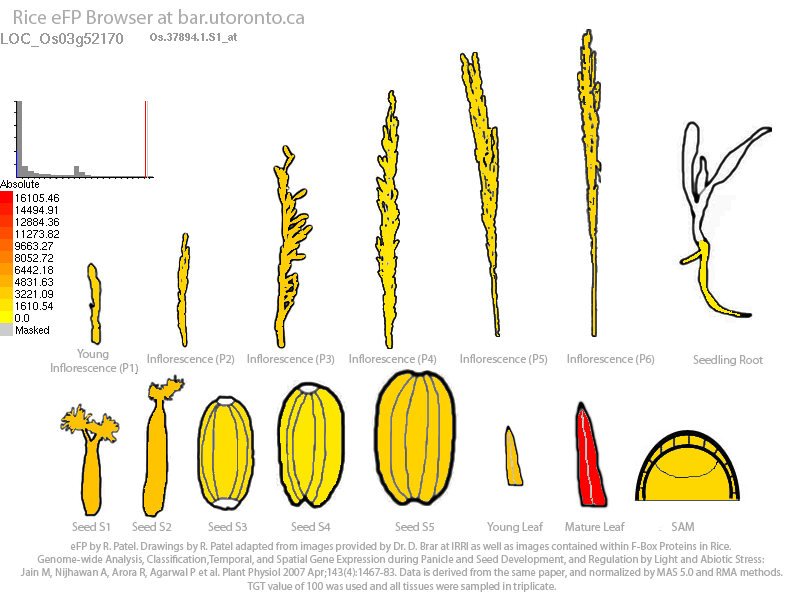
**

Supplemental Figure 2. Expression pattern of *OsHMBPP* at different growth stages. Data was collected from the Rice eFP Browser.

**
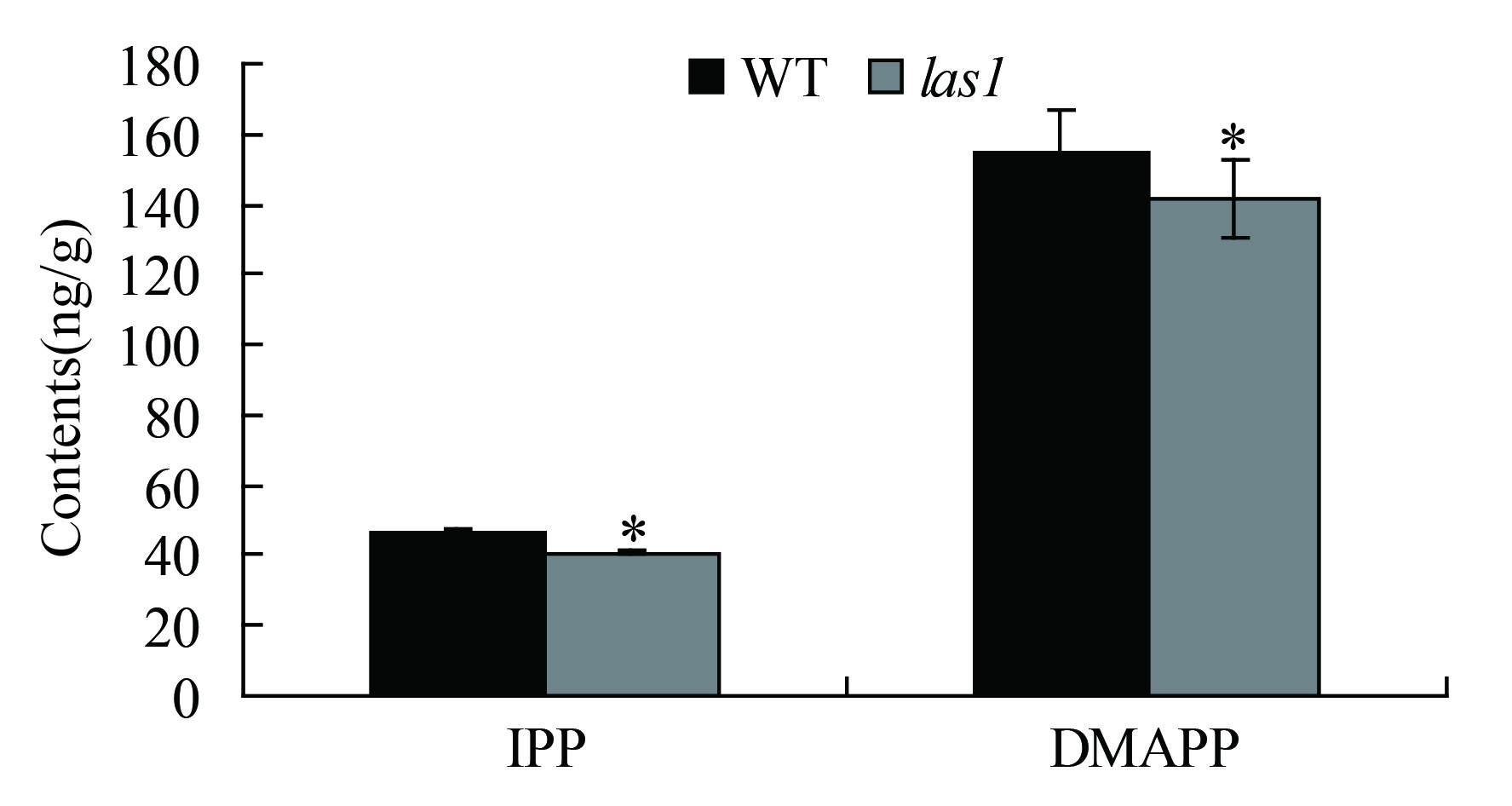
**

Supplemental Figure 3. Determination of IPP and DMAPP between WT and *las1*.

**
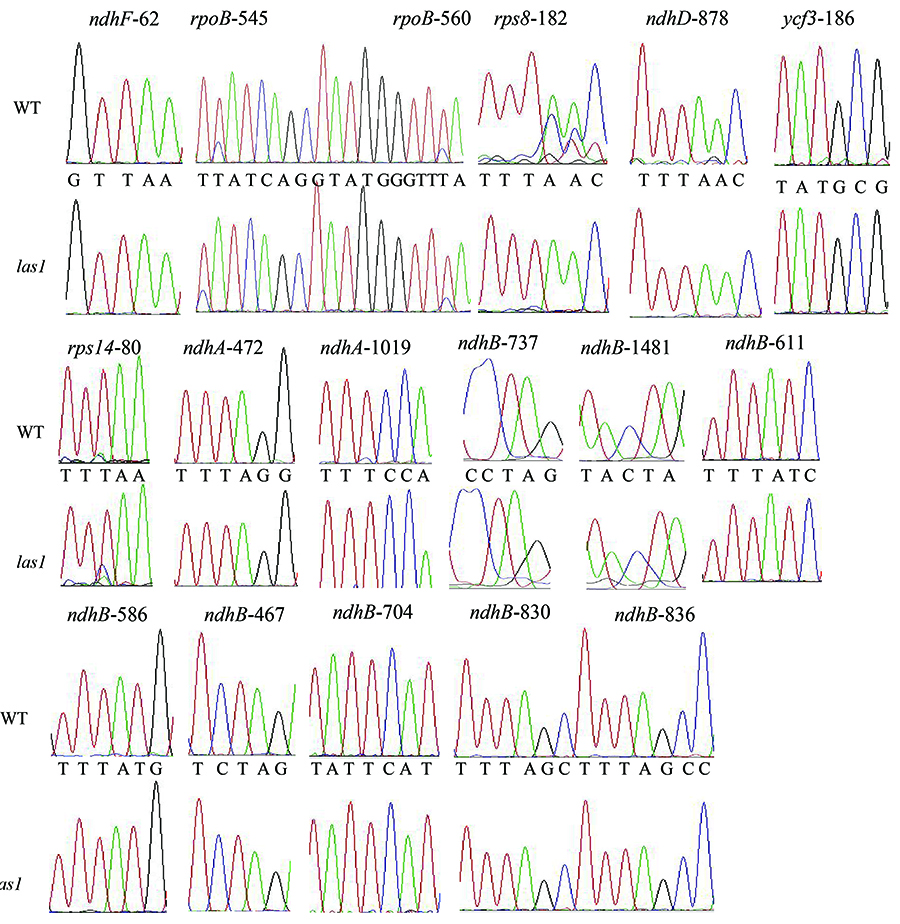
**

Supplemental Figure 4. The rest 17 chloroplast RNA editing sites between WT and *las1*.

**
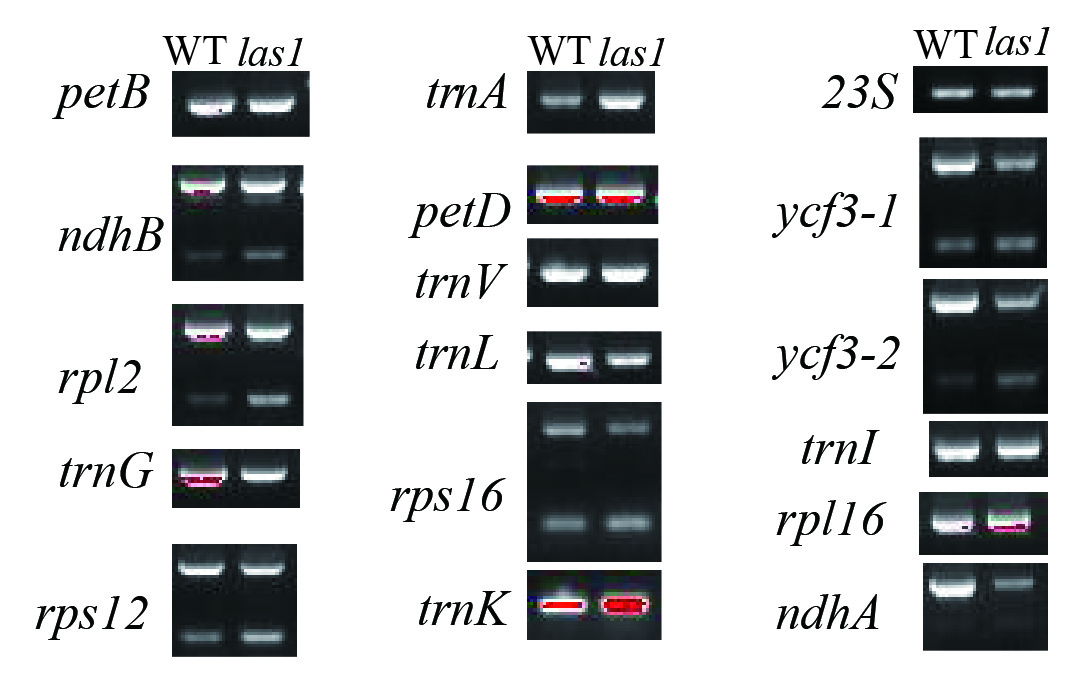
**

Supplemental Figure 5. Splicing Analysis of rice chloroplast genes in wild-type and *las1*.

**
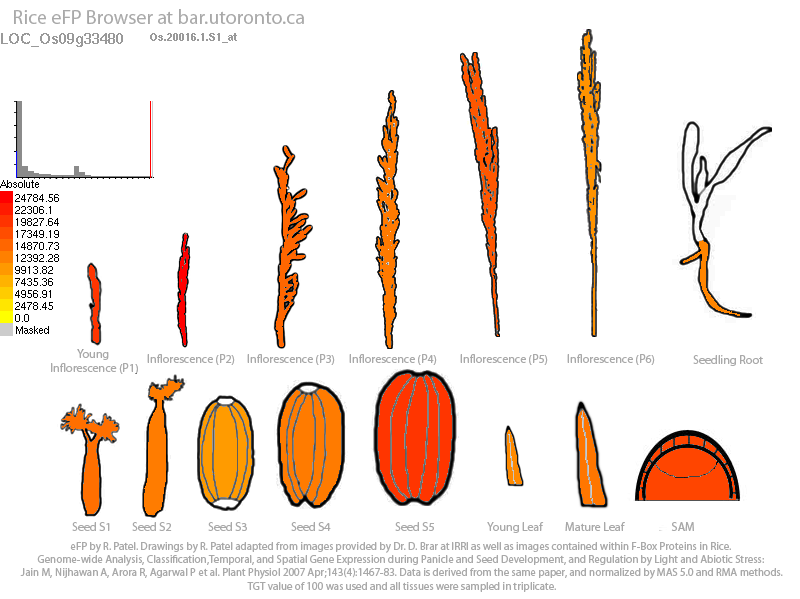
**

Supplemental Figure 6. Expression pattern of [*Os09g33480*](http://rice.plantbiology.msu.edu/cgi-bin/ORF_infopage.cgi?orf=LOC_Os09g33480) at different growth stages. Data was collected from the Rice eFP Browser.

**
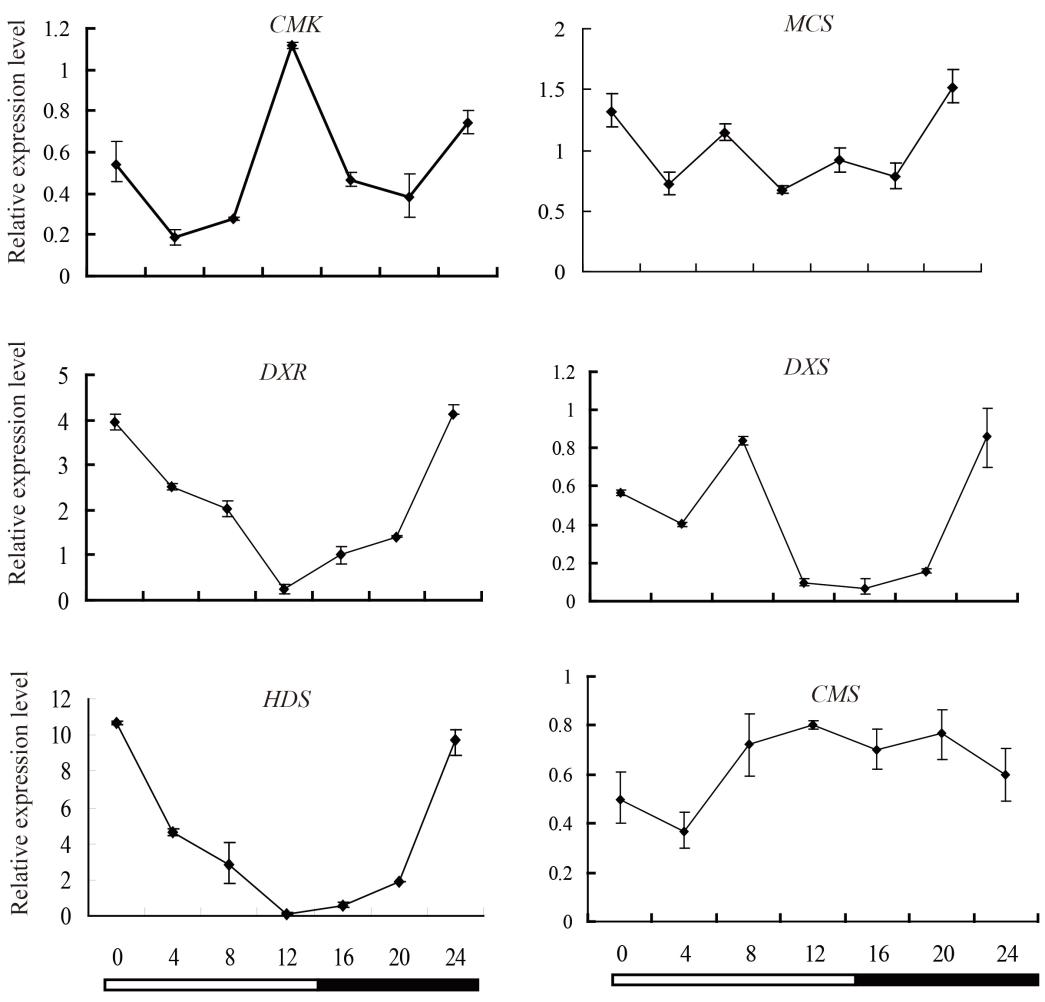
**

Supplemental Figure 7. Expression patterns of rice nonMVA pathway genes in a 14h light/ 10h dark. Wild-type seedlings were grown in a growth chamber for 14 days after germination, and then the seedlings were sampled every 4 h. *Error bars* indicate SD (n= 3).

**
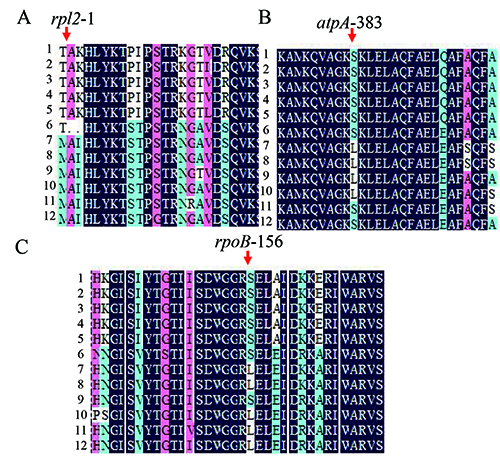
**

Supplemental Figure 8. RNA-edited amino acids alignment of the plastid rpl2, atpA and rpoB in monocot(1-6) and eudicot(7-12) . 1: Zea mays, 2: Oryza sativa, 3: Hordeum vulgare, 4: Sorghum bicolor, 5:Triticum aestivum, 6: Phoenix dactylifera, 7: Arabidopsis thaliana, 8:Brassica napus, 9: Nicotiana tabacum, 10: Glycine max, 11: Vitis vinifera, 12: Gossypium arboreum.

Supplemental Table 1 Chloroplast signal peptide prediction

| chlorop v1.1 prediction | | | | | |
| --- | --- | --- | --- | --- | --- |
| Name | Length | Score | cTP | CS-score | cTP-length |
| LAS1 | 459 | 0.561 | Y | 7.733 | 31 |

"Y" means that the sequence is predicted to contain a cTP. CS-score is the MEME scoring matrix score for the suggested cleavage site. cTP-length is the predicted length of the presequence.

Supplemental Table 2 Primers used in real-time PCR and vector construction

| Marker | Forward primer sequence (５′ →３′ ) | Revers primer sequence （５′ →３′ ） |
| --- | --- | --- |
| Crispr-LAS1 | GGCATGGCGACCATCACCACGCAGCTC | AAACGAGCTGCGTGGTGATGGTCGCCA |
| LAS1g | ACTCCGAATCCGACGCACCC | TTCATAATCACCTTGTTGACG |
| pAN580-LAS1 | CGGAGCTAGCTCTAGAATGGCGACCATCACCACGCA | TGCTCACCATGGATCCCGCGGCCTGCAAAACTTC |
| qLAS1 | GCGAGCTTGTCGAGAAAGAG | GCGCTTGATCTCAAACACCT |
| qDXS | TCTTCCACGTCTCCAAGACC | CTTGTGAGGGTACGACTGGT |
| qDXR | AAACGAGGGACAGAAGAGCA | GAACCGGTTGAGCCAACAAT |
| qCMK | AAGGAGCTTCAGGAGTGGTC | GCATGCTTCAGGTGGCTTTA |
| qHDS | TGTGGCCAAGAGCTCAAGTA | TGGGATGATCACTGCCAAGT |
| qMCS | GGATCCATCCGTCGTCAATC | TGAGCAGCTATGCTCCTGTT |
| qCMS | AAACGGGATGGACTTGAGGT | CTCATTCATCAGGCGCTCAG |
| UBQ | AGACCACCAAGTACTACTGCAC | CCACCGATCTTGTACACGTCC |
| OsHMBPP-nYFP | CATTTACGAACGATAGTTAATTAAATGGCGACCATCACCACGCA | CACTGCCACCTCCTCCACTAGTCGCGGCCTGCAAAACTTC |
| Os09g33480-cYFP | CATTTACGAACGATAGTTAATTAAATGGCGTCGGCGTCGCGCTT | CACTGCCACCTCCTCCACTAGTCTGGTAATTCCTCCCTT |
